# New Medical Device and Therapeutic Approvals in Otolaryngology: State of the Art Review of 2022

**DOI:** 10.1002/oto2.105

**Published:** 2024-01-22

**Authors:** Franklin M. Wu, Daniel Gorelik, Michael J. Brenner, Masayoshi Takashima, Amit Goyal, Ashley E. Kita, Austin S. Rose, Robert S. Hong, Waleed M. Abuzeid, Peter S. Maria, Ahmed A. Al‐Sayed, Michael E. Dunham, Prajoy Kadkade, Scott R. Schaffer, Alan W. Johnson, Adrien A. Eshraghi, Shireen Samargandy, Robert J. Morrison, Philip A. Weissbrod, Margaret B. Mitchell, Cyrus C. Rabbani, Neil Futran, Omar G. Ahmed

**Affiliations:** ^1^ Department of Otolaryngology–Head and Neck Surgery Houston Methodist Hospital Houston USA; ^2^ Department of Otolaryngology–Head & Neck Surgery University of Michigan Medical School Ann Arbor USA; ^3^ Department of Otorhinolaryngology All India Institute of Medical Sciences Jodhpur Jodhpur USA; ^4^ Department of Head and Neck Surgery David Geffen School of Medicine at UCLA Los Angeles USA; ^5^ University of North Carolina School of Medicine Department of Otolaryngology–Head and Neck Surgery; ^6^ Michigan Ear Institute Farmington Hills USA; ^7^ Department of Otolaryngology–Head and Neck Surgery Wayne State University Detroit USA; ^8^ University of Washington Department of Otolaryngology–Head and Neck Surgery; ^9^ Stanford University Department of Otolaryngology–Head and Neck Surgery; ^10^ King Saud University Department of Otolaryngology–Head & Neck Surgery; ^11^ Louisiana State University Health Sciences Center School of Medicine Department of Otolaryngology–Head and Neck Surgery; ^12^ Columbia University–Harlem Hospital Department of Surgery; ^13^ Department of Surgery NYU Long Island School of Medicine New York City USA; ^14^ Department of Otorhinolaryngology–Head and Neck Surgery Hospital University of Pennsylvania Philadelphia USA; ^15^ Department of Otolaryngology–Head & Neck Surgery Park Nicollet Specialty Care Bloomington USA; ^16^ Department of Otolaryngology and Neurosurgery University of Miami Miller School of Medicine Miami USA; ^17^ Department of Otolaryngology–Head and Neck Surgery University of Arizona Tucson USA; ^18^ Department of Otolaryngology–Head and Neck Surgery King Abdulaziz University Jeddah Saudi Arabia; ^19^ Division of Otolaryngology–Head and Neck Surgery University of California San Diego La Jolla USA; ^20^ Department of Otolaryngology–Head & Neck Surgery Harvard Medical School/Mass Eye and Ear Boston USA; ^21^ Department of Otolaryngology–Head and Neck Surgery Case Western Reserve University and University Hospitals Cleveland USA

**Keywords:** innovation, medical review, new devices

## Abstract

**Objective:**

To review new drugs and devices relevant to otolaryngology approved by the Food and Drug Administration (FDA) in 2022.

**Data Sources:**

Publicly available FDA data on drugs and devices approved in 2022.

**Review Methods:**

A preliminary screen was conducted to identify drugs and devices relevant to otolaryngology. A secondary screen by members of the American Academy of Otolaryngology–Head and Neck Surgery's (AAO‐HNS) Medical Devices and Drugs Committee differentiated between minor updates and new approvals. The final list of drugs and devices was sent to members of each subspecialty for review and analysis.

**Conclusion:**

A total of 1251 devices and 37 drugs were identified on preliminary screening. Of these, 329 devices and 5 drugs were sent to subspecialists for further review, from which 37 devices and 2 novel drugs were selected for further analysis. The newly approved devices spanned all subspecialties within otolaryngology. Many of the newly approved devices aimed to enhance patient experience, including over‐the‐counter hearing aids, sleep monitoring devices, and refined CPAP devices. Other advances aimed to improve surgical access, convenience, or comfort in the operating room and clinic.

**Implications for Practice:**

Many new devices and drugs are approved each year to improve patient care and care delivery. By staying up to date with these advances, otolaryngologists can leverage new innovations to improve the safety and quality of care. Given the recent approval of these devices, further studies are needed to assess long‐term impact within the field of otolaryngology.

Medical innovation presents opportunities for improving existing therapies, and a wide range of medical technology and therapeutics are continually being developed and brought to market to advance care and address unmet needs. The Food and Drug Administration (FDA) oversees the approval of new medical devices and drugs. This state‐of‐the‐art review evaluates devices and drugs approved in 2022.

## Methods

This review adhered to previously described methodology for assessing new FDA approvals.^1^, ^2^, ^3^ All medical devices and drugs that received FDA approval from January 1 to December 31, 2022 were eligible for inclusion in this review. The FDA provides publicly available databases of records related to approvals for de novo devices, premarket approvals, and 510(k) submissions. Records reviewed by specific committees of the FDA including ENT (ear, nose, and throat), neurosurgery, anesthesia, plastic surgery, and general surgery were assessed along with newly approved drugs. Two independent reviewers completed the initial review of each approval. Further screening and final evaluation for each device and drug was completed by committee members with corresponding subspecialty expertise, assessing novelty, relevance, and impact for the specialty. Companies were contacted for clarification of product details as needed and for permission to reproduce images of products.

## Results

**Figure 1:**
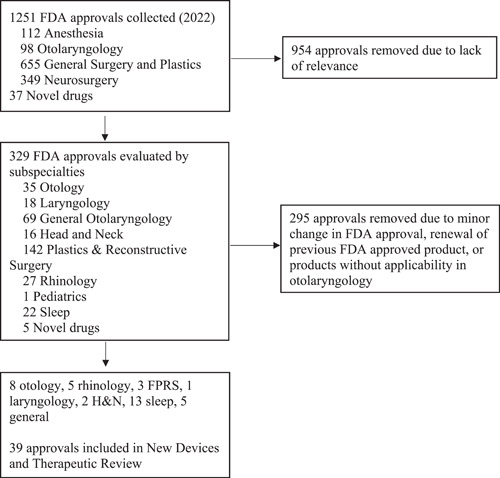
Flowchart of process used to review and determine new Food and Drug Administration‐approved devices and drugs in 2022.

A review of 2022 FDA‐approved drugs and devices was conducted. Among these, 112 anesthesia, 98 otolaryngology, 655 general surgery and plastics, and 349 neurosurgery devices were identified and collected, and 37 new drugs approvals were also identified (Figure 1). From this group, 954 drugs and devices were removed from an initial screen due to lack of relevance to the field of otolaryngology. From the remaining 329 devices and drugs, 295 were removed in a secondary screen conducted by specialists within otolaryngology. A final list of 37 devices and 2 novel drugs were identified and reviewed by members of the AAO‐HNS Medical Devices and Drugs Committee (Table 1).

**Table 1:** Otolaryngology‐Relevant Devices and Drugs Approved by the Food and Drug Administration in 2022 by Subspecialty

	Devices (n = 37)	Drugs (n = 2)
Otology	Over‐The‐Counter Hearing Aids (6)	None
	OtoSight Middle Ear Scope	
	OTOPLAN	
Rhinology	TruDi Shaver Blade	None
	RhinAer Stylus	
	Stryker Intrao‐Operative OR Hub (2)	
	VISIONAIR	
Plastic & Reconstructive Surgery	MIRIA Skin Treatment System	DAXXIFY
		NexoBrid
Laryngology	Phagenyx Neurostimulation System	None
Head and Neck/Endocrine	Vesseal	None
	da Vinci E‐200 Surgical Generator	
Sleep	Sleep Monitoring devices (8)	None
	Sunrise	
	SomnoMetry	
	CPAP Devices (4)	
Pediatrics	Electric Nasal Aspirator	None
General	AG100s	None
	Ultrasonic Surgical System	
	Ultrasonic Scalpel System	
	MolecuLightDX	
	ENT‐Flex™ Rhinolaryngoscope	

## Discussion

### Otology

#### Over‐the‐Counter (OTC) Hearing Aids

In August of 2022, the FDA issued a final rule that established a new category of OTC hearing aids. This allowed individuals 18 years and older with mild‐to‐moderate hearing loss to purchase hearing aids directly from retailers, without the assistance of a hearing health care professional. It provides a regulatory framework for OTC hearing aids, including device design requirements (such as mandating a user‐adjustable volume control), safety features (such as providing limits on the maximum sound output), and performance specifications. This rule is intended to both expand access and lower the cost of hearing aids.

While purchase of OTC devices does not require a diagnostic hearing test, OTC hearing aids are not appropriate for individuals with severe or profound hearing loss or those younger than 18 years old, who still require prescription hearing devices. Several devices received FDA approval in the past year, including: Vibe SF (WSAUD A/S), Nuheara IQbuds 2 PRO Hearing Aid (Nuheara Limited), MDHearing Smart Hearing Aid, Jabra Enhance Plus (GN Hearing A/S), Eargo Self‐Fitting Hearing Aid (Eargo, Inc. and BHA100 Series Braun Clear Hearing Aid (Kaz USA, Inc.). These FDA changes could allow individuals suffering from hearing loss to have earlier interventions and assistance while they wait for full evaluation with an audiologist and otolaryngologist, potentially reducing the morbidity associated with hearing loss.

#### Otosight Middle Ear Scope

The OtoSight Middle Ear Scope (PhotoniCare) is a video otoscope which allows for direct visualization of the tympanic membrane with optical coherence tomography. The technology also allows physicians to view beyond the tympanic membrane to assess for middle ear pathology.^4^ It uses low‐coherence light to capture 2D and 3‐dimensional (3D) images of the middle ear tissue. The technology allows clinicians to visualize past cerumen, other canal obstructions, and opacified tympanic membranes to guide the clinician in evaluating middle ear disease.^5^, ^6^ The technology uses low‐coherence light to capture 2D and 3D images of the middle ear tissue.

#### OTOPLAN®

**Figure 2:**
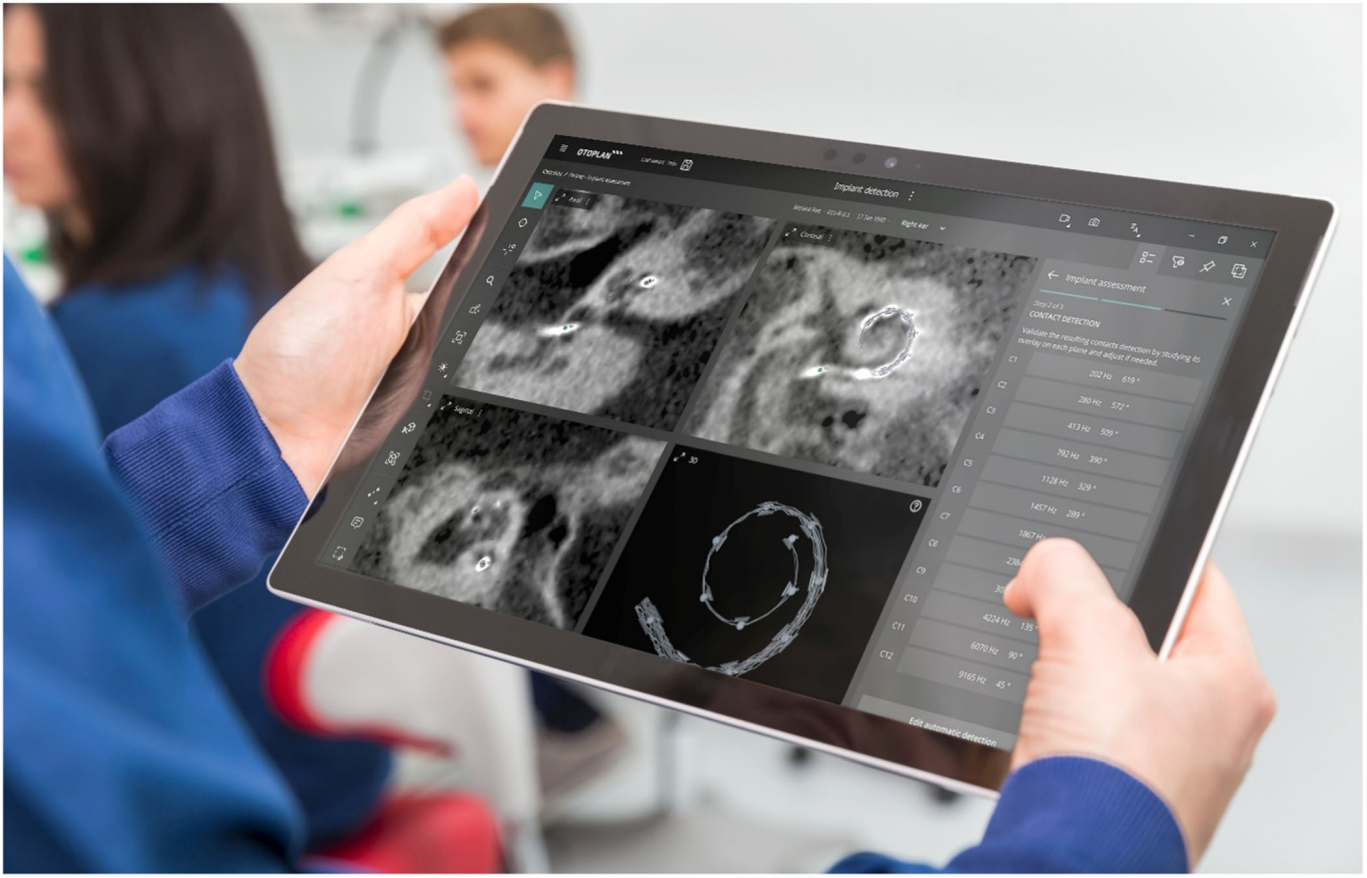
The OTOPLAN® surgical planning software.

OTOPLAN® was developed in close collaboration between MED‐EL and CASCINATION AG, and the technology provides a full, 3D reconstruction of the temporal bone to facilitate surgical planning in cochlear implantation surgery (Figure 2). OTOPLAN features automatic calculation of preoperative cochlear parameter measurements, which allows surgeons to select electrode arrays according to the specific cochlear anatomy duct length. It also allows surgeons to visualize the potential electrode array position post‐op implantation. The software creates reconstructions of the cochlea, round window membrane, bony overhang, semi‐circular canals, and internal auditory canal. The software uses image collection from both magnetic resonance imaging and computed tomography scans aiming to provide the most accurate preview of the insertion depth and frequency coverage of each electrode array.^5^, ^6^ The software can also give postoperative feedback to visualize and perform a quality check of the insertion and lead management status. The product has shown success in selection of appropriate CI electrodes in pediatric inner ear malformations.^7^, ^8^ It has been proposed that the new version of the software may also be used for BONEBRIDGE placements.

### Rhinology

#### TruDi Shaver Blade

**Figure 3:**
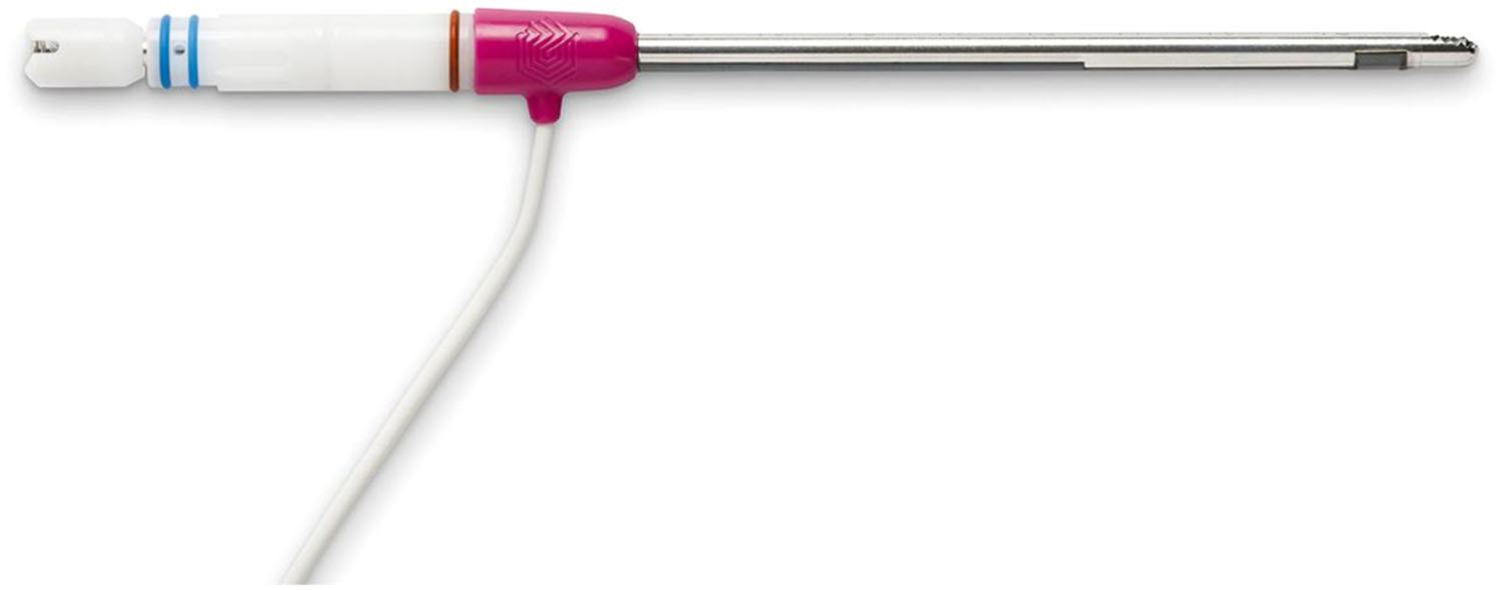
The TruDi® Shaver Blade.

The TruDi Shaver Blade (Acclarent Inc.) is a single‐use, electromagnetically navigated blade intended for use with the Bien‐Air S120 Shaver handpiece and OSSEODUO or ORiGO powered systems (Le Noirmont) (Figure 3). The blade, available in several configurations, allows for the incision and removal of both soft tissue and bone in otolaryngology and oral‐maxillofacial surgery. The device includes 3 and 4 mm straight blades and 4 mm blades with 15°, 40°, and 60° angles. This new shaver blade is also designed for use with the TruDi Navigation System (version 2.3 or later) for 3D image‐guided surgery, with distal tip electromagnetic sensor integration. This device offers surgeons another option for a tissue shaver with image guidance capabilities.

#### RhinAer Stylus

**Figure 4:**
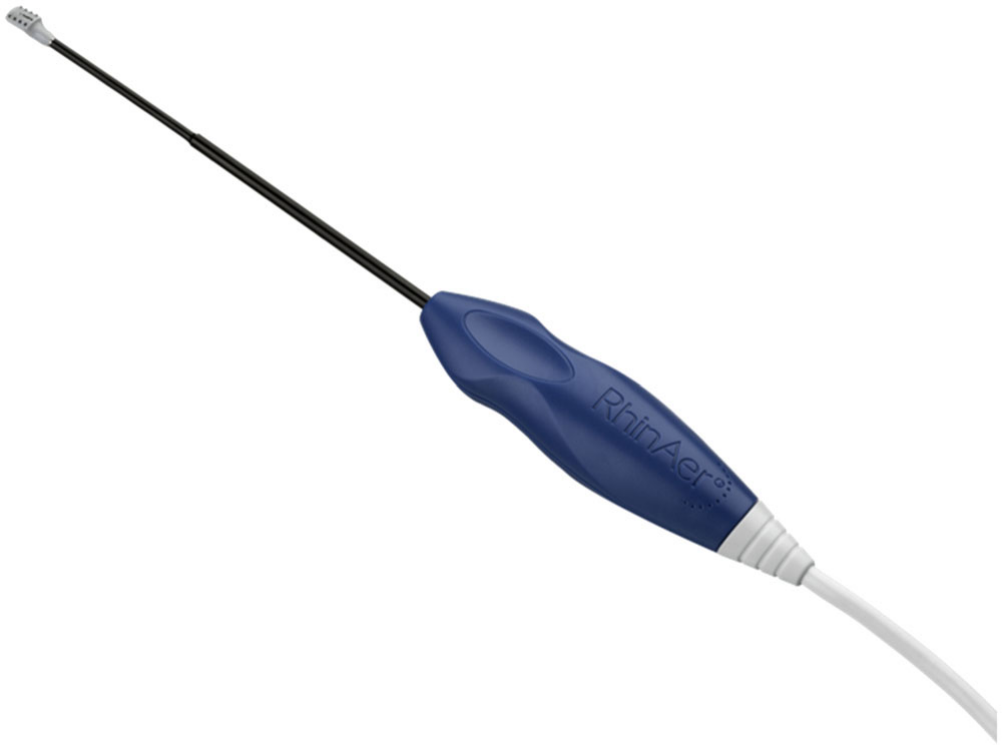
The new RhinAer® stylus.

The RhinAer stylus (RhinAer) is an office‐based probe used to deliver bipolar radiofrequency energy to tissue in the nasal passages, including areas around the posterior nasal nerve, to treat patients with chronic rhinitis symptoms (Figure 4). There are multiple prospective studies including a randomized clinical trial showing improvement in chronic allergic and non‐allergic rhinitis symptoms compared to placebo 1 year after treatment.^9^, ^10^, ^11^, ^12^ The current version incorporates modifications to the stylus that do not change its function, but improve surgeon access and visualization. The updated model has a slimmer shaft and a 10° downward tilt of the tip to minimize visual obstruction and improve access to the posterior middle meatus, particularly in patients with unfavorable anatomy.^13^ Further studies to assess efficacy with the new design are needed. This new design will be particularly helpful for clinicians in patients with narrow/obstructive nasal anatomy. The new design will help clinicians maneuver the device around septal deviations, lateral nasal side walls, and more inflamed/edematous mucosa.

#### Stryker's Intra‐Operative OR Hub

In 2022, Stryker released updates to their intraoperative OR control systems. The SDC4K Information Management System with Device and Voice Control Package is a new version of Stryker's integrative intraoperative system which allows surgeons and operating room staff to control devices in a hands‐free manner with voice commands. The updated version includes a new console with inputs and outputs in HDMI format. These systems allow for greater efficiency during procedures, removing the need to stop surgical progress to adjust certain features. These devices can also capture, store, and display medical device data. The 1688 4K Camera Control Unit with Advanced Imaging Modality 89 and 1688 4K Pendulum Camera Head is also part of Stryker's intraoperative OR Hub. These devices include a camera head and control unit for use with endoscopes or laparoscopes. The devices incorporate a sensor for the camera head, allowing it to rotate the image for the surgeon to maintain orientation. While the literature has not explored the utility of this device, it may be useful in rhinologic or otologic procedures utilizing multiple devices and may have benefits in surgical training. However, these devices require institutional buy‐in for a fully interconnected system.

#### VISIONAIR™

VISIONAIR™ Rhinoanemometer by PacificMD Biotech is a software application that aims to provide a faster and noninvasive alternative to traditional rhinometry methods such as acoustic rhinometry and anterior rhinomanometry. It uses light reflection technology to map the size and structure of the nasal airway and applies an artificial intelligence algorithm to measure the cross‐sectional area of the nasal valve and cavum from endoscopic images. The system consists of a Windows 10‐based smart device, algorithm, and user interface for data entry and display, with the option of storing data locally or to the cloud, while patient information is encrypted and stored anonymously. Although the literature is limited, the device offers a standardized and objective approach to assessing nasal airway patency, which can help clinicians decide on potential surgical candidates and improve quantification of outcomes following interventions for nasal airway obstruction.^14^

### Plastic & Reconstructive Surgery

#### MIRIA Skin Treatment System®

MIRIA Skin Treatment System® (AVAVA Inc.) is an intra‐dermally focused 1550 nm laser for skin resurfacing and soft tissue coagulation. Clinical indications include aging skin, wrinkles, sun damage, pseudofolliculitis barbae, and deep acne scarring. The MIRIA System's Focal Point Technology delivers up to 150 mJ of conical energy to the dermis and thus differs from conventional cylindrical lasers. The conical configuration enables clinicians to focus energy delivery to specific depths of the skin, thereby theoretically enhancing the safety across different skin types and pigmentation. Lasers target chromophores in the skin, and melanin can be an unintended chromophore. With traditional lasers, darker skin and higher melanin concentration increase the risk for excess energy absorption, posttreatment hyperpigmentation, and loss of pigment.^15^ Patients with darker skin types treated with a 1550 nm laser have increased risk of post‐inflammatory hyperpigmentation.^16^ The MIRIA Skin Treatment System aims to minimize such risks by focusing the laser energy to a clinician‐specified skin depth, allowing a larger maximum pulse energy than predicates (150 vs 70 mJ). Clinical trials are needed to establish if MIRIA's conical technology is superior to EpilME® (2022) or LightSheer® Desire (2017) lasers, which also have FDA clearance for all skin types (Fitzpatrick I‐IV).

#### Anacaulase‐bcdb (NexoBrid®)

NexoBrid® (MediWound), is a bromelain‐based (a group of enzymes found in the fruit and stem of the pineapple plant) proteolytic enzyme product that is applied for eschar debridement in adult patients with deep partial or full‐thickness thermal burns. Surgical debridement, which reduces bacterial colonization, risk of infection, and nonvital tissue, is the standard of care for burns injuries. However, NexoBrid®, approved in Europe since 2013, is a nonsurgical option for rapid, selective debridement.^17^ It has been validated in a multicenter randomized clinical trial and the phase 3 DETECT trial.^18^, ^19^ It achieved ≥95% wound closure rate, shorter time to eschar removal versus gel vehicle, and lower blood loss versus surgery. NexoBrid was noninferior to surgery in cosmesis and function at 12 and 24 months. Utilization of NexoBrid® in the head and neck has been studied in facial burn injuries. In comparisons of NexoBrid® versus surgical debridement for deep facial burns, NexoBrid® significantly improved scar quality, decreased the number of procedures needing complete debridement, and reduced size of autografting necessary after debridement.^19^ At 1 year of follow‐up, scar quality was improved in the NexoBrid® group compared to surgical debridement. Outcomes in scar quality included pigmentation, pliability, stiffness, thickness, surface area, and scar irregularity. A 2020 European consensus statement highly recommended enzymatic debridement in facial burns with protection of the eyes and facial orifices, further increasing the potential impact of NexoBrid within otolaryngology.^17^

#### DaxibotulinumtoxinA‐lanm DAXXIFY™

DAXXIFY™ (Revance Therapeutics Inc.) is a long‐acting neuromodulator that is FDA‐approved in adults for injection to improve moderate to severe glabellar lines from corrugator and/or procerus muscle activity. DAXXIFY™ provides up to double the duration of abobotulinumtoxinA (BOTOX®/BOTOX® Cosmetic), abobotulinumtoxinA (DYSPORT®), incobotulinumtoxinA (XEOMIN®), rimabotulinumtoxinB (MYOBLOC®), and prabotulinumtoxinA‐xvfs (JEUVEAU®). DAXXIFY® contains botulinum toxin type A, which blocks presynaptic acetylcholine release at the neuromuscular junction, with reversible chemical denervation of muscle. It incorporates Peptide Exchange Technology™ (PXT) to stabilize the neuromodulator, allowing prolonged duration of action. Unlike other neuromodulators, it contains no human or bovine serum albumin. DAXXIFY™ was evaluated in the SAKURA study program, which enrolled more than 2700 patients who underwent approximately 4200 treatments.^20^, ^21^, ^22^, ^23^ In randomized, double‐blind, placebo‐controlled trials, onset was typically evident within 2 days, and 98% of subjects achieved none or mild wrinkle severity at 4 weeks with a 6‐month median duration of defect.^20^, ^21^ In open‐label phase 3 safety studies, no serious side effects were noted, and most commonly observed adverse reactions were headache (6%), eyelid ptosis (2%), and facial paresis (1%).^22^, ^23^

### Laryngology

#### Phagenyx® Neurostimulation System for Rehabilitation of Oropharyngeal Dysphagia

**Figure 5:**
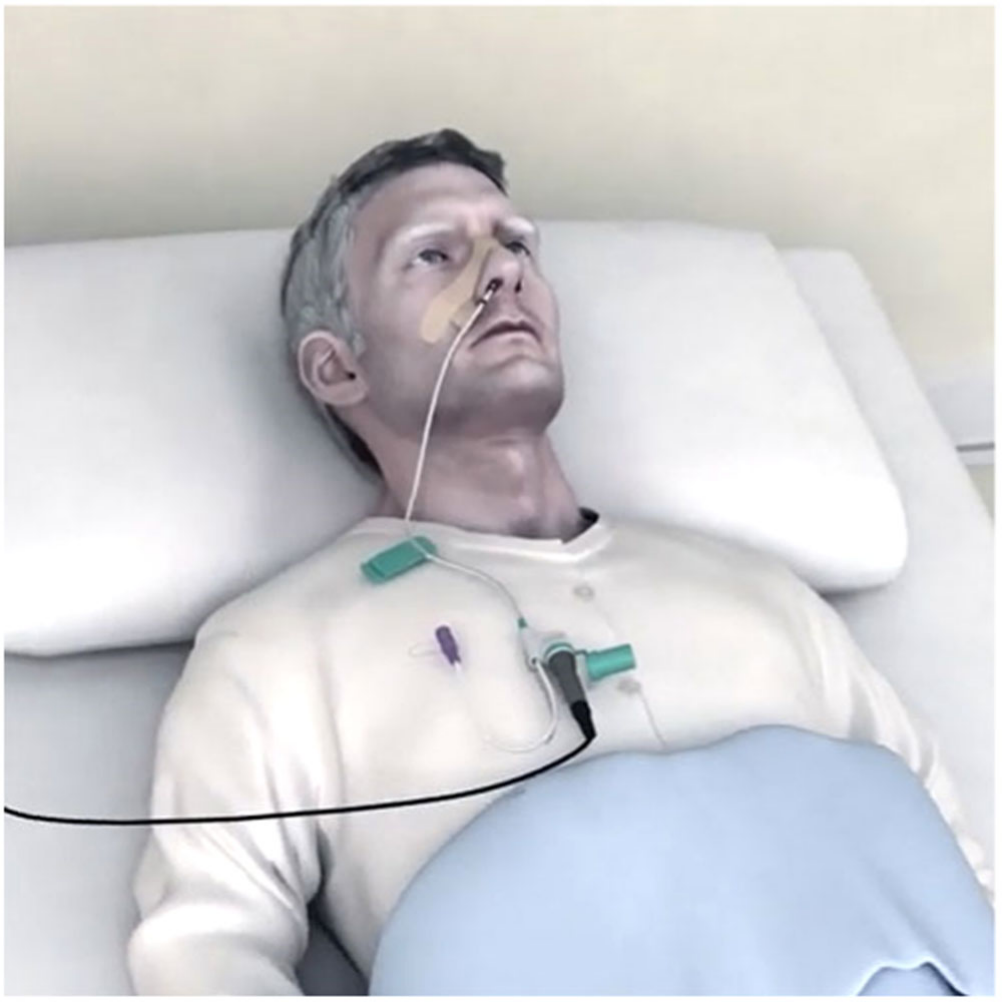
Illustration of Phagenyx® pharyngeal electrical stimulation catheter in place, which can also be used to administer enteral nutrition during treatment.

The Phagenyx*®* Neurostimulation System (Phagenesis Limited) is a device which uses pharyngeal electrical stimulation (PES) to improve pharyngeal swallow function. The device is based on research demonstrating that sensory stimulation results in motor cortex re‐organization.^24^ The system involves placement of a soft hollow catheter through the nose, advanced through the pharynx to the stomach. The catheter then serves as enteral access for nutrition, while the proximal portion contains electrodes for pharyngeal electrical stimulation (Figure 5). Stimulation is delivered for 10 minutes a day and therapy is recommended for 3 to 6 days. Early randomized controlled studies have demonstrated pharyngeal electrical stimulation may be associated less radiographic aspiration and clinical dysphagia,^25^ which has been replicated in a prospective single‐arm observational cohort with the Phagenyx device in a neurogenic dysphagia patient population.^26^ This may have potential use in our head and neck cancer population post‐treatment but further research is needed.

### Head and Neck/Endocrine

#### Vesseal^TM^

Vesseal^TM^ (Lydus Medical) is an automated microvascular anastomosis suture deployment system. The needle insertion mechanism contains two‐rod protrusions intended to locate, mount, and position the blood vessel relative to the stopper mechanism. Each rod stores 8 curved needles that deploy double‐armed nylon sutures. The handle mechanism features right and left switches to deploy the needles on the respective rod side. The delivery needle is taper‐pointed stainless steel which can be used for end‐to‐end anastomosis with sutures ranging from United States Pharmacopeia (USP) sizes 7‐0 to 9‐0. There are similar devices available in the market such as Perclose ProGlide^TM^.

#### Da Vinci E‐200 Electrosurgical Generator

The da Vinci E‐200 Electrosurgical Generator (Intuitive) allows surgeons to precisely cut, coagulate, and remove tissue using high‐frequency electrical currents in robotic and other minimally invasive surgical procedures.^27^ Electrosurgical generators are commonly used in minimally invasive surgeries such as robotic surgery, including transoral procedures for head and neck cancer.^28^ Efficient energy instrumentation is crucial for endoscopic minimally invasive surgery. The E‐200 Electrosurgical Generator is compatible with a broad range of instruments. It offers multiple high‐frequency settings for monopolar and bipolar modes, which can be used for tissue cutting, coagulation, and desiccation. It also has an advanced bipolar radiofrequency mode that is compatible with the da Vinci SynchroSeal and Vessel Sealer Extend advanced energy instruments.

### Sleep

#### Improvements and Innovations in Sleep Monitoring for Home Sleep Studies

**Figure 6:**
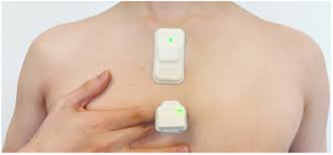
ANNE sleep system worn on sternum and finger.

This past year's FDA‐approved developments in sleep apnea technology focused on improving home sleep assessment and improving CPAP compliance. This area is becoming increasingly important for Otolaryngologists who are playing a bigger role in the management of OSA with advent of hypoglossal nerve stimulators. Studies have shown 20% to 50% of single‐night sleep testing results in misclassification of OSA severity.^29^ Therefore, new at‐home technologies allow multi‐night testing for capturing the potential night‐to‐night variability. Among these devices, the Cerebra Sleep System (Cerebra Medical Ltd.), WatchPAT300 (Itamar Medical Ltd.), Nomad PMU810 (Neurotronics), RespiraSense (PMD Solutions), NightOwl (Ectosense NV), BresoDX1 (Bresotec Inc.), Onera Sleep Test System (Onera BV), and ANNE Sleep System (Sibel Inc.) (Figure 6) offer improvements in data collection, patient comfort, ease of use, and convenience. Many studies have been conducted to validate their accuracy,^30^, ^31^, ^32^, ^33^, ^34^, ^35^, ^36^ but further research is required to assess performance compared to other devices on the market.

**Figure 7:**
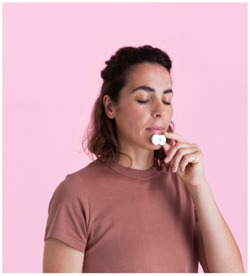
Sunrise device worn on the anterior chin.

Several devices incorporate artificial intelligence (AI) for measuring and calculating respiratory effort for at‐home sleep studies. Sunrise (Sunrise SA) uses AI‐powered software along with the measurement of jaw movements through a disposable lightweight 6‐channel device that fits on the chin (Figure 7). Results recently published showed that mandibular jaw movement captured with the Sunrise system is a highly reliable and noninvasive surrogate of the esophageal pressure as the current gold‐standard to quantify respiratory effort during sleep.^37^, ^38^, ^39^

#### SomnoMetry

(Neumetry) is an autoscoring artificial intelligence/machine learning (AI/ML) software which automatically analyzes and scores sleep study results. While the technology automates a portion of a sleep study, the results should still be verified and analyzed by a trained physician.

#### Innovations in CPAP devices

To improve CPAP compliance, devices have been improved to be more portable with reduced noise. These improvements include intelligent auto‐adjustment and humidifier integration with wireless ways to retrieve data (Auto CPAP System by BMC Medical Co., Ltd.), quieter and more portable BiPAP with leak auto‐compensation (Luna G3 BPAP System by 3B Medical Inc.), masks that are more form‐fitting, moldable, and low profile which allows for optimum mask seal with minimum tension (AirGel Technology with IQ 2 Phantom 2 Nasal Mask by Sleepnet Corp.), as well as stability wings to ensure the masks stay on (Evora Full Face Mask by Fisher & Paykel Health care Ltd.).

### Pediatrics

#### Electric Nasal Aspirator

The electric nasal aspirator (Shenzhen XinLianFeng Technology Company, LTD.) is a portable device used to suction excess nasal secretions in children ages 2 to 12. One iteration of the product, which is commercially available, includes 3 levels of suction and an incorporated music function, with adjustable volume, to help distract and calm young children during use. This is one of many devices available that can help improve nasal airflow and decrease nasal congestion in the pediatric population. This device is useful for parents, but it is important for otolaryngologists to be aware of these technologies. There appear to be potential applications for this new device in the maintenance of nasal hygiene as well as improved management of clinical nasal rhinorrhea, congestion, and obstruction.

### General

#### AnapnoGuard AG100s Cuff Pressure and Airway Management System

The AG100s (Hospitech Respiration Ltd.) monitors and controls the cuff pressure of endotracheal and tracheostomy tubes and evacuates secretions from the subglottic space above the cuff during mechanical ventilation. This device automatically maintains cuff pressure pre‐set by the user or based on monitoring of carbon dioxide pressure above the cuff. If used with a tube from Hospitech, it can also perform intermittent evacuation of subglottic secretions from above the tube's cuff. The AG100s FDA approval was expanded to include the ability to connect the system to tracheostomy tubes. It is designed to be used with the AnapnoGuard ET tube. This tube helps to address known problems of cuff pressure monitoring and secretion management to avoid complications from tracheostomy tubes.^40^ While limited in its portability, the device helps maintain appropriate cuff pressures in patients requiring mechanical ventilation. This has the potential to decrease rates of subglottic and tracheal stenosis and aspiration if pressures are set appropriately below 30 cmH_2_O.

#### Ultrasonic Surgical Devices

There are two new ultrasonic surgical dissection devices. The Ultrasonic Scalpel System from SurgNova Health Technologies, is intended for transection, dissection, and ligation soft tissue. This product encompasses 4 components: an energy generator, footswitch, handpiece, and multiple scalpel blades ranging in length from 13 to 43 cm. The UltraSonic Surgical System from Miconvey Technologies Co., Ltd. updates their prior ultrasonic scalpel; this product has similar components, although its scalpel blades range from 1.4 to 4.5 cm and offer additional functions, such as grasping in addition to cutting and coagulating tissue. Compared to other instruments which can cut and cauterize tissue, products utilizing ultrasonic energy may have reduced thermal damage and stronger coagulation properties and allow surgeons to seal vessels up to 8 mm. While described more commonly in laparoscopic literature,^41^ these devices have been used in head and neck procedures as well, including thyroidectomy, free flap harvest, tonsillectomy, and transoral laryngeal cancer resections.^42^, ^43^, ^44^, ^45^ Data comparing ultrasonic devices to traditional bipolar and cold‐steel instruments are limited.

#### MolecuLightDX^TM^

**Figure 8:**
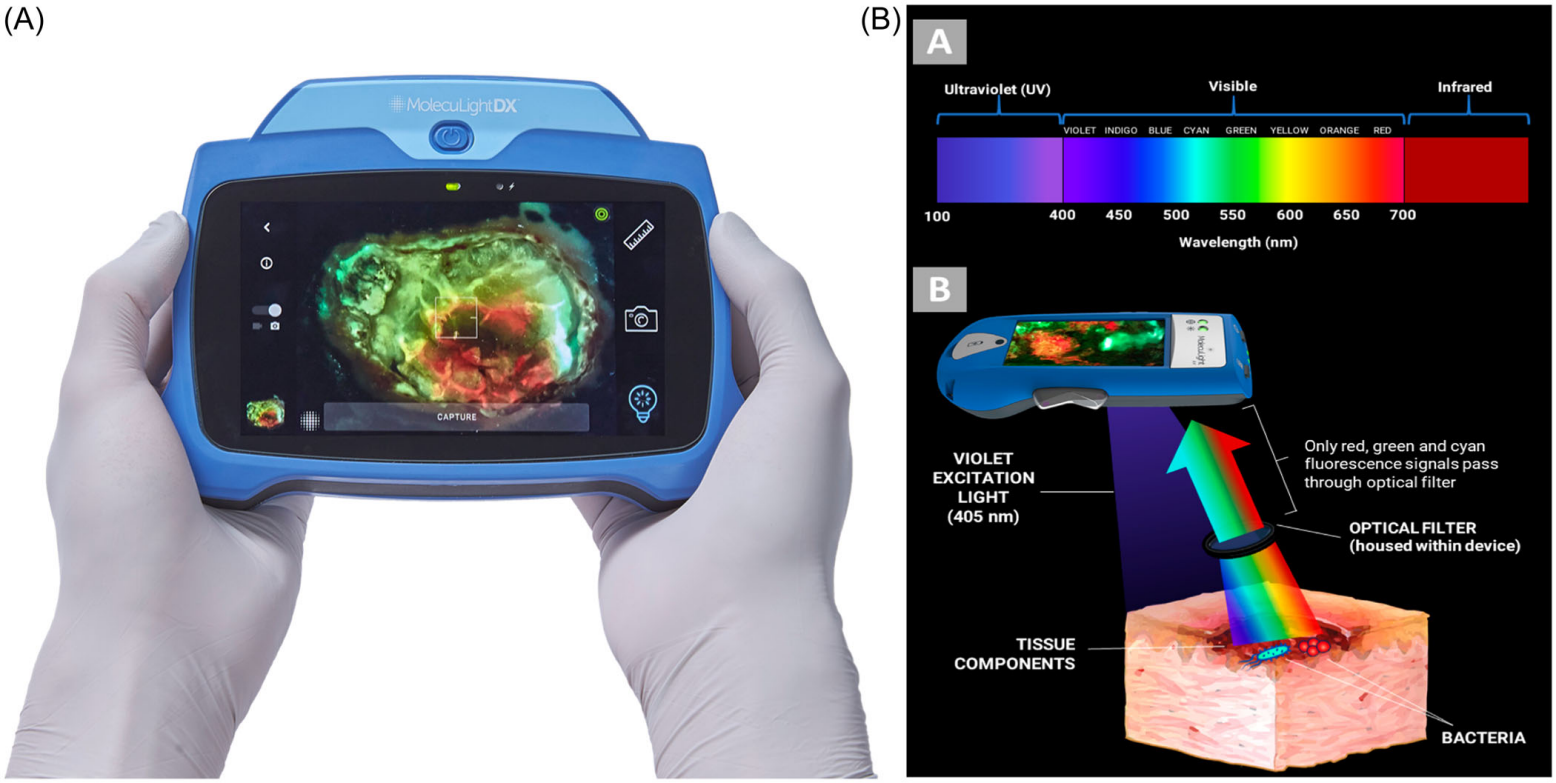
(A) MolecuLightDX^TM^ fluorescence image scanning system. (B) Optical filtering mechanism to only record specific wavelengths of fluorescence signals.

MolecuLightDX^TM^ is a fluorescence imaging scanning system that allows the examination and photography of skin wounds, using fluorescence to predict elevated bacterial loads (Figure 8). Many studies of this technology have focused on healing of diabetic foot ulcers, although it has also been shown to improve healing in other wounds. It might facilitate the evaluation and treatment of nonhealing wounds, burns, and trauma of the head & neck. The system allows clinicians to take accurate digital wound measurements to allow for greater precision for monitoring wounds.^46^, ^47^, ^48^, ^49^

#### ENT‐Flex™ Rhinolaryngoscope

**Figure 9:**
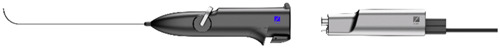
The ENT‐Flex™ Rhinolaryngoscope with the single‐use shell and reusable imaging core.

The ENT‐Flex™ Rhinolaryngoscope (Zsquare) is a single‐use flexible endoscope featuring a reusable imaging core with a disposable sterile outer shell involving the handle, steering mechanism, and steerable shaft (Figure 9). This scope uses a newly designed single polymeric fiber which allows a smaller optical diameter without compromising resolution. Other endoscopes use complementary metal oxide semiconductor (CMOS) technology with a chip in the distal tip, which limits size and resolution. This new single fiber technology allows for a slim 2.3 mm outer diameter scope, the smallest in the market. This adds to the growing market of single‐use endoscopes, which theoretically reduces the risk of cross‐contamination and obviates the need for processing or repair costs. This device has not been formally evaluated in the literature. However, the ENT‐Flex™ may provide a cost‐effective option by offering the outer component with a handle for the physician, effectively providing a barrier for the reusable imaging core while eliminating the need for autoclaving or discarding the entire device.

## Limitations

This study was subject to limitations. While a panel of experts within each subspecialty were consulted to determine which products and drugs should be included in the study, these decisions can be subjective, and there may be disagreements on which devices/drugs should and should not have been discussed. Furthermore, the potential impact of many of the devices and drugs previously discussed could not be fully elucidated due to the lack of relevant literature currently available. The full impact of many of these innovations will likely not be fully understood until many years later once further studies have been published.

## Implications for Practice

The field of otolaryngology continued to grow and advance in 2022. As otolaryngology progresses, clinicians need to stay abreast of advances in new devices and drugs to optimize care of their patients.

## Author Contributions

**Franklin M. Wu**, data collection, data analysis, manuscript writing, manuscript revisions; **Daniel Gorelik**, data collection, data analysis, manuscript writing, manuscript revisions; **Michael J. Brenner**, data analysis, manuscript writing, manuscript revisions; **Masayoshi Takashima**, data analysis, manuscript writing, manuscript revisions; **Amit Goyal**, data analysis, manuscript writing, manuscript revisions; **Ashley E. Kita**, data analysis, manuscript writing, manuscript revisions; **Austin S. Rose**, data analysis, manuscript writing, manuscript revisions; **Robert S. Hong**, data analysis, manuscript writing, manuscript revisions; **Waleed M. Abuzeid**, data analysis, manuscript writing, manuscript revisions; **Peter S. Maria**, data analysis, manuscript writing, manuscript revisions; **Ahmed A. Al‐Sayed**, data analysis, manuscript writing, manuscript revisions; **Michael E. Dunham**, data analysis, manuscript writing, manuscript revisions; **Prajoy Kadkade**, Data analysis, manuscript writing, manuscript revisions; **Scott R. Schaffer**, data analysis, manuscript writing, manuscript revisions; **Alan W. Johnson**, data analysis, manuscript writing, manuscript revisions; **Adrien A. Eshraghi**, data analysis, manuscript writing, manuscript revisions; **Shireen Samargandy**, data analysis, manuscript writing, manuscript revisions; **Robert J. Morrison**, data analysis, manuscript writing, manuscript revisions; **Philip A. Weissbrod**, data analysis, manuscript writing, manuscript revisions; **Margaret B. Mitchell**, data analysis, manuscript writing, manuscript revisions; **Cyrus C. Rabbani**, data analysis, manuscript writing, manuscript revisions; **Neil Futran**, data analysis, manuscript writing, manuscript revisions; **Omar G. Ahmed**, study design, data collection, data analysis, manuscript writing, manuscript revisions.

## Disclosures

### Competing interests

Waleed M. Abuzeid, MD, Consultant for Medtronic and Acclarent (Johnson & Johnson). Michael E. Dunham, MD, MS, Partner at Soteria Medical Solutions. Alan W. Johnson, MD, MS, Consultant for Invisian Medical, LLC and receives patent royalties from the University of Minnesota for dental occlusion ties/Minne Ties. Neil Futran, MD, DMD, Educational consultant for Stryker Corporation. Adrien A. Eshraghi, MD, MSc, Research grant from MEDEL GmBH and SoundPharma through the University of Miami.

### Funding source

None.
